# A survey of proteomic biomarkers for heterotopic ossification in blood serum

**DOI:** 10.1186/s13018-017-0567-2

**Published:** 2017-05-04

**Authors:** Laura E. Edsberg, Erin L. Crowgey, Patrick M. Osborn, Jennifer T. Wyffels

**Affiliations:** 1 Center for Wound Healing Research, Natural Sciences, Daemen College, Amherst, NY 14226 USA; 2 Bioinformatics, Nemours Biomedical Research, Nemours Alfred I. duPont Hospital for Children, Wilmington, DE 19803 USA; 3 San Antonio Military Medical Center, San Antonio, TX USA; 4 Center for Bioinformatics & Computational Biology, University of Delaware, Newark, DE 19711 USA

**Keywords:** Heterotopic ossification, SRM-MS, Osteocalcin, Osteomodulin, Collagen alpha-1(v)

## Abstract

**Background:**

Heterotopic ossification (HO) is a significant problem for wounded warriors surviving high-energy blast injuries; however, currently, there is no biomarker panel capable of globally characterizing, diagnosing, and monitoring HO progression. The aim of this study was to identify biomarkers for HO using proteomic techniques and blood serum.

**Methods:**

Isobaric tags for relative and absolute quantitation (iTRAQ) was used to generate a semi-quantitative global proteomics survey of serum from patients with and without heterotopic ossification. Leveraging the iTRAQ data, a targeted selection reaction monitoring mass spectrometry (SRM-MS) assay was developed for 10 protein candidates: alkaline phosphatase, osteocalcin, alpha-2 type I collagen, collagen alpha-1(V) chain isoform 2 preprotein, bone sialoprotein 2, phosphatidate phosphatase LPIN2, osteomodulin, protein phosphatase 1J, and RRP12-like protein.

**Results:**

The proteomic survey of serum from both healthy and disease patients includes 1220 proteins and was enriched for proteins involved in the response to elevated platelet Ca^+2^, wound healing, and extracellular matrix organization. Proteolytic peptides from three of the ten SRM-MS proteins, osteocalcin preprotein, osteomodulin precursor, and collagen alpha-1(v) chain isoform 2 preprotein from serum, are potential clinical biomarkers for HO.

**Conclusions:**

This study is the first reported SRM-MS analysis of serum from individuals with and without heterotopic ossification, and differences in the serum proteomic profile between healthy and diseased subjects were identified. Furthermore, our results indicate that normal wound healing signals can impact the ability to identify biomarkers, and a multi-protein panel assay, including osteocalcin preproprotein, osteomodulin precursor, and collagen alpha-1(v) chain isoform 2 preprotein, may provide a solution for HO detection and monitoring.

**Electronic supplementary material:**

The online version of this article (doi:10.1186/s13018-017-0567-2) contains supplementary material, which is available to authorized users.

## Background

Heterotopic ossification (HO) is the formation of mature lamellar bone in nonosseous (soft) tissues [1]. HO has been associated with war injuries since World War I and is now recognized as a significant comorbidity for wounded warriors surviving high-energy blast injuries [1–3]. A study of combat-related extremity injuries in Operation Enduring Freedom (OEF) and Operation Iraqi Freedom (OIF) found the risk of HO is highest following a blast mechanism injury and an amputation within the zone of injury [1]. HO in the military population often results in chronic pain, difficulties fitting prostheses, joint ankylosis, functional limitations, prolonged rehabilitation, and substantial morbidity [4, 5]. Rates of HO in combat-related extremity injuries are greater than 60% [1, 2].

In civilian populations, HO may occur after a traumatic event, including hip arthroplasty, distal humerus fractures, spinal cord injuries (SCI), and closed brain injuries [6]. The etiology of HO remains unknown, but clinical risk factors include trauma, amputation, traumatic brain injury (TBI), SCI, thermal injury, major hip arthroplasty, and other major orthopedic surgery [7]. The exact cellular events leading to HO are not yet identified and as a result, treatment has been limited to nonsteroidal anti-inflammatory (NSAID) drugs and local radiation therapy used prophylactically. Many patients require one or more surgical excisions of ectopic bone [7]. Individuals with combat injuries often have additional diagnoses for which these prophylactic treatments are contraindicated further limiting treatment options. NSAIDs can cause severe gastrointestinal problems, renal toxicity, and platelet deficiency. Similarly, radiation therapy carries significant risks including fracture nonunion, genetic mutation, malignancy, and reproductive organ damage. No current pharmaceutical treatment is approved by the FDA to treat HO [7].

The etiology of HO is not well characterized. Aberrant bone growth associated with the disease is diagnosed using radiographs. In order to prevent the development of HO, early detection of biomarkers associated misregulated wound healing mechanisms is necessary. Serum is readily available and easily accessible for repeated sampling for biomarker identification, and robust serum biomarkers have been established for other disorders, including cardiovascular disease [8]. The development of HO requires a cell capable of bone production, an osteoinducive factor, and an environment supportive of osteoinduction [7]. Researchers have evaluated changes in proteins associated with osteoinduction in the serum following traumatic brain injury (TBI) in rats and humans [9], and serum from TBI patients accelerated the proliferation of osteoblastic differentiation in cells from human muscle [10]. In contrast to blood serum, tissue biopsy relative to the lesion samples are invasive and location of the biopsy can impact results. Collectively, this work supports the use of serum for the identification of markers associated with the development of HO.

Recent advancements in mass spectrometry (MS) technology have enabled proteomic analysis of complex biological samples and have aided in the identification of potential biomarkers in various diseases, including evaluation of bone metabolism [11]. A high-throughput MS technique, isobaric tags for relative and absolute quantitation (iTRAQ), enables a global analysis of the proteome differences between biological samples, which provides the foundation for identifying potential biomarkers, but additional quantitative assays are required. Proteomic differences identified using iTRAQ are expressed as a ratio between samples and are therefore relative and semi-quantitative. There are several quantitative assays available for protein biomarker analysis, such as antibody approaches used in ELISAs or peptide approaches used in advanced mass spectrometry assays. Antibody-based approaches are limited based on reagent specificity and availability, whereas a targeted MS assay is limited only by the proteolytic and ionization characteristics of the protein of interest. One type of MS assay, selection reaction monitoring (SRM-MS), is an advanced proteomics technology enabling the identification and precise quantification of peptides with high sensitivity, specificity, and reproducibility [12, 13]. Analytical information from peptides obtained using SRM-MS allows by inference, quantification of the corresponding proteins in complex biological samples. This technique is capable of producing high-quality diagnostic data in disease processes [14] and is a more sensitive and reproducible method for quantifying low-abundance proteins in complex biological samples. SRM-MS uses synthetic peptides to optimize detection transition parameters for each peptide target and as such is not an appropriate method for a high-throughput proteomics analysis. The objectives of this study were to develop an SRM-MS assay specific for overexpressed proteins present in the serum of subjects with HO and test their predictive ability using serum from subjects with and without HO.

## Methods

### Subject enrollment

This study was part of a larger project to study the proteomics of HO in tissue and serum (manuscript in preparation), focusing on mass spectrometry analyses of serum samples from 41 subjects. Subjects were eligible for enrollment in the study if they had or were being treated for high-risk fractures, acetabular fracture, burns with orthopedic injury, traumatic brain injury with extremity trauma, undergoing amputation, and excision of ectopic bone or major arthroplasty. Subjects below the age of 18 or currently being treated for cancers or metastatic disease involving the bone were excluded.

### Study protocol and overview

**Fig. 1:**
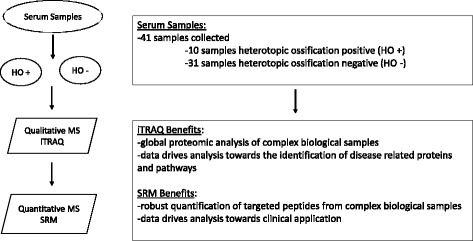
Sample processing overview. A total of 41 serum samples were collected at time of surgery from subjects with (*n* = 10) and without (*n* = 31) heterotopic ossification. Serum samples were pooled by disease state and subjected to an isobaric tag for relative and absolute quantitation (iTRAQ) mass spectrometry (MS) analysis. The iTRAQ data was used to drive the selection of specific proteins to target via a selected reaction monitoring (SRM) MS technique, a qualitative MS technique that enables the robust quantification of specific peptides within a single subject’s serum sample

Participants were enrolled prior to surgery, and outcome was determined by evaluation of x-rays collected at the time of surgery and during follow-up visits at 6 weeks, 12 weeks, 6 months, and 12 months. Blood (5 cm³) was collected at time of the patient’s scheduled surgical procedure. Blood samples were collected into sterile vacutainer tubes with clot activator and gel for serum separation. Serum was removed after 30 min to a sterile screw top polypropylene tube and snap frozen in liquid nitrogen before storage at −80 °C. Samples were assigned to HO-positive or HO-negative group-based disease status at the time of surgery and blood collection. Blood serum samples were pooled in equal volumes by disease status and analyzed using iTRAQ. Individual serum samples were analyzed for the abundance of specific proteins identified in the iTRAQ results using SRM-MS, a targeted quantitative analysis (Fig. 1).

### Sample and peptide preparation for iTRAQ

Reagents were purchased from Sigma-Aldrich (St. Louis, MO) unless otherwise indicated. The top 14 most abundant proteins in the serum were depleted using the Seppro IgY14 column systems: albumin, IgG, α2-antitrypsin, IgA, IgM, transferrin, haptoglobin, α2-macroglobulin, fibrinogen, complement C3, α1-acid glycoprotein (orosomucoid), HDL (apolipoproteins A-I and A-II), and LDL (mainly apolipoprotein B). Serum samples were diluted 5× in IgY dilution buffer, filtered (0.22 μm), and then injected into IgY LC10 columns attached to an Agilent 1200 HPLC system. The unretained fraction was collected.

In-solution depleted serum samples were further processed by MyOmicsDx, Inc (Towson, MD, USA) using “filter-assisted sample preparation” (FASP) method [15]. Briefly, protein samples in 9 M urea were reduced with 5 mM tris-(2-carboxyethyl) phosphine (TCEP) at 37 °C for 45 min and reduced cysteines were blocked using 50 mM IAA at 25 °C for 15 min. Protein samples were cleaned using 10 kDa Amicon Filter (UFC501096, Millipore) three times using 9 M urea and two times using MyProt-Buffer 1 (MyOmicsDx, Inc). Samples were proteolyzed with trypsin (V5111, Promega) for 12 h at 37 °C.

The peptide solution was acidified by adding 1% trifluoroacetic acid (TFA) and incubated at room temperature for 15 min. A Sep-Pak light C18 cartridge (Waters Corporation) was activated by loading 5 mL 100% (vol/vol) acetonitrile and washed by 3.5 mL 0.1% TFA solution two times. The acidified digested peptide solution was centrifuged at 1800 × *g* for 5 min, and the supernatant loaded into the cartridge. To desalt the peptides bound to the cartridge, 1, 3, and 4 mL of 0.1% TFA were used sequentially. To elute the peptides from the cartridge, 2 mL of 40% (vol/vol) acetonitrile with 0.1% TFA was used. The eluted peptides were lyophilized overnight and reconstituted in 37 μL MyProt-Buffer 3 (MyOmicsDx, Inc, Towson, MD, USA).

### Multiplexed iTRAQ labeling

Digested peptides from samples in a volume of 37 μl MyProt-Buffer 2 were labeled using 4-plex iTRAQ reagents (ABSciex, Framingham, MA, USA). After 2 h, labeled peptides were dried to remove organic solvents and reconstituted in 500 μl MyProt-Buffer 3 (MyOmicsDx, Inc, Towson, MD, USA), combined and fractionated on a bRPLC (basic reverse phase liquid chromatography) column (XBridge BEH C18 Column, 5 μm, 2.1 × 100 mm) via XBridge BEH C18 Guard Column (Waters Corporation) using an Agilent 1260 HPLC system. Peptides in each fraction were dried and re-suspended in 8 μl 0.1% formic acid (EMD Millipore, Billerica, MA, USA) with 3% acetonitrile for LC-MS/MS analysis.

A Sep-Pak light C18 cartridge (Waters Corporation) was activated by loading 5 mL 100% (vol/vol) acetonitrile (JT Baker) and was washed by 3.5 mL 0.1% TFA solution two times. Acidified digested peptide solution was centrifuged at 1800*g* for 5 min, and the supernatant was loaded into the cartridge. To desalt the peptides bound to the cartridge, 1, 3, and 4 mL of 0.1% TFA were used sequentially. To elute the peptides from the cartridge, 2 mL of 40% (vol/vol) acetonitrile with 0.1% TFA was used, and this elution was repeated two more times for a total of 6 mL of eluate. It was important to ensure that the cartridge had stopped dripping before each sequential wash and elution solution was applied. The eluted peptides were lyophilized overnight and reconstituted in 37 μL of MyProt-Buffer 2 (MyOmicsDx, Inc, Towson, MD, USA).

### Nanoflow electrospray ionization tandem mass spectrometry analysis

Data-dependent MS/MS analyses of the iTRAQ-labeled peptides were carried out by MyOmicsDx, Inc. (Towson, MD) on a Q Exactive™ Hybrid Quadrupole-Orbitrap Mass Spectrometer (https://www.thermofisher.com/us/en/home.html) interfaced with Proxion nanoflow LC system. Peptides were fractionated by reverse phase HPLC on a 75 μm × 15 cm PicoFrit column packed with Magic C18AQ (5 μm, 120 Å, https://www.bruker.com/) using 0–60% acetonitrile/0.1% formic acid gradient over 90 min at 300 nL/min. Eluting peptides were sprayed directly into Q Exactive™ at 2.0 kV.

Survey scans (full MS) were acquired from 350 to 1800 m/z with up to 15 peptide masses (precursor ions) individually isolated with a 2-Da isolation window and fragmented (MS/MS) using a collision energy of 29% and 30 s dynamic exclusion. Precursor and the fragment ions were analyzed at 70,000 and 17,500 resolutions, respectively. Peptide sequences were identified from isotopically resolved masses in MS and MS/MS spectra extracted with and without de-convolution using Thermo Scientific MS2 processor and Xtract software.

### iTRAQ-MS data processing

Mass spectrometry raw files were automatically processed through Proteome Discoverer 2.1 software using Xtract and MS2-processor spectrum processor in addition to default spectrum selector node. The data was searched in Refseq 2015 human entries using Mascot search engine interfaced with different processing nodes of Proteome Discoverer 2.1. Search parameters included oxidation on methionine, iTRAQ 4-plex on tyrosine, deamidation on residues N and Q as different variable modifications, iTRAQ 4-plex on N-terminus and lysine residue, and methylthio on cysteine residue as different fixed modifications. Mass tolerances on precursor and fragment masses were set to 15 ppm and 0.03 Da, respectively. Peptide validator node was used for peptide validation with stringent cutoff of 0.01 and relaxed cutoff of 0.05 false discovery rate (FDR), and 1% FDR cutoff was used to filter the data.

High confidence (0.1% FDR) and top ranked peptides were considered with protein grouping options. Protein ratios were normalized through MyProt-QuantiR (MyOmicDx, Inc) software package, and peptides with >30% isolation interference were excluded from protein quantification to avoid potential interference of reporter ions from contaminant peaks. MA plots were used to evaluate any potential bias between quantification channels within experiment and between experiments.

### Proteomic bioinformatics analysis

The entire iTRAQ dataset, regardless of protein ratio, was uploaded into Cytoscape v3.3.0 [16] and analyzed using the ReactomeFI plugin (database 2015) [17] to generate an interactome, followed by a pathway enrichment analysis. Gene ontology enrichment analysis for biological process, cellular component, and molecular function were completed using the cytoscape app BiNGO [18] and REVIGO [19]. The iTRAQ ratio (expression) data was overlaid with biological annotations to prioritize proteins for SRM analysis.

### Reagents SRM-MS

TCEP (tris-(2-carboxyethyl) phosphine) was purchased from Thermo Scientific (Waltham, MA). LysC and trypsin proteases were purchased from Promega (Fitchburg, WI). C18 Cartridges for sample preparation and chromatography columns for bRPLC and online HPLC of Triple Quadrupole mass spectrometer were purchased from Waters (Milford, MA). Acetonitrile was purchased from JT Baker, and formic acid was obtained from EMD Millipore (Billerica, MA, USA). MyProt-Buffer 1, MyProt-Buffer 2, and MyProt-Buffer 3 were utilized by MyOmicsDx, Inc (Towson, MD, USA). All other reagents were purchased from Sigma-Aldrich (St. Louis, MO) unless otherwise indicated.

### SRM-MS data processing

Peptide samples reconstituted in 37ul MyProt-Buffer 3 (MyOmicsDx, Inc) were spiked with *MyProt-SRM Internal Control Mixture* (MyOmicsDx, Inc) composed of a pool of 1 f mole heavy isotope-labeled peptides covering a large hydrophobicity window and a large M/z range (M/z 200 ~ 1300) and were subject to SRM analysis. Peptide samples were eluted through an online Agilent 1290 HPLC system into the Jet Stream ESI source of an Agilent 6495 Triple Quadrupole Mass spectrometer.

Thirty peptides representing 10 proteins were chosen as SRM targets from MyOmicsDx’s manually curated SRM target peptide database, *MyProt-SRM Map*, based on their iTRAQ ratio. Transition parameters and retention times of the 30 peptides were confirmed individually using an Agilent 6495 Triple Quadrapole Mass Spectrometer for both doubly and triply charged precursor ions. Five or 6 transitions per peptide precursor were selected for SRM analysis.

Three hundred and fifty sets of transition parameters (corresponding to 30 peptides, representing the abundance of 10 proteins) and 30 SRM data files containing the quantitative data of 30 peptides in 30 human serum samples (Additional file 1) were imported into Skyline 3.1 [20]. The abundance of each target peptide was represented by the total area under the curve (AUC) of all its transitions normalized to the total AUC of all transitions from the most nearby (sharing a similar hydrophobicity) heavy isotope-labeled peptide from MyProt-SRM Internal Control Mixture (MyOmicsDx, Inc) spiked in before the SRM analysis. The abundance of each target peptide was represented by the total AUC of all its transitions normalized to the total AUC of each control peptide’s transitions. The relative abundance level of a target peptide in different samples was represented by its relative ratio to the abundance level of the internal control peptide in the same sample. The abundance dataset was further normalized by the ratio obtained from a subject without HO, (ND-1), chosen randomly as a reference disease negative sample in this study. Differences in SRM peptide abundance were tested using R and Welch’s *t* test.

### Model construction

The dataset obtained from the SRM-MS assay was composed of 30 samples, 10 HO positive and 20 HO negative, with 17 peptide abundance values for each sample. The number of samples in this pilot study was limited, and the number of parameters (*p*) for each subject (*n*) is relatively large compared to the total number of samples (*n* = 30). The SRM assay dataset was analyzed using three models, random forest (RF), generalized linear model (GLM), and support vector machine learning (SVM).

Random forest, originally proposed by Breiman in 1999, is an ensemble classification algorithm composed of a series of decision trees. Each tree is built independently through a technique called “bagging” based on random selection of input variables. The prediction result is based on the vote made by all trees. This modeling approach provides a very accurate classifier, but it has not been widely used in clinical diagnostics. In contrast, logistic regression is a widely used standard regression model for binary data. It has been widely used to construct biomarker panels for clinical diagnostics. Support vector machine learning is a supervised machine learning model that identifies the optimal separating hyperplane between two classes or states based on least-squares regression of the data.

Seventy percent of the dataset was used to construct models, and thirty percent of the dataset was withheld to evaluate model performance. Resampling, regression, and prediction were repeated 1000 times for each model. Model performance was evaluated by comparing the results generated from at least 100 predictions for each of the three models, and the subset of models used to do the prediction in each category were preselected with a AUC of ROC no smaller than 0.98.

## Results

### iTRAQ results

#### Proteomics analysis and serum biomarker selection

In total, 41 subjects including 27 men and 14 women ranging in age from 22 to 83 were enrolled (Table 1). HO-negative samples were derived largely from total hip arthroplasty (ages 28–83) in both male and female subjects ages 22–83. All HO-positive samples were collected during HO excision or hip revision procedures in men and women ages 22–40. Serum samples were analyzed via qualitative proteomics analysis and/or a targeted quantitative analysis (Fig. 1). The high-throughput proteomics analysis consisted of a 4-plex iTRAQ experiment, which utilized separately pooled serum samples from HO-positive (*n* = 10) and HO-negative (*n* = 31) subjects. No bias between biological or technical replicates was observed, and median normalization was applied to the raw data to allow direct comparison of the reported ratios of the proteins between HO-positive and HO-negative groups. Collectively, 1220 unique proteins (UniProtKB Accession) were measured, and a ratio per protein was calculated between separately pooled HO-positive and HO-negative serum samples. The majority of the proteins had an expression ratio between 0.5 and 1.5 (data not shown).

**Table 1:** Surgical procedures and heterotopic ossification status of serum samples

	HO negative				HO positive			
	Subjects	M	F	Age	Subjects	M	F	Age
All	31	18	13	22–83	10	9	1	22–40
Total hip arthroplasty (THA)	18	10	8	28–83	–			
Open reduction and internal fixation (ORIF)	7	5	2	26–64	–			
Hip revision (HR)	3		3	45–62	2	1	1	36–40
HO excision (HOE)	–				8	8		22–31
Other	3	3		22–36	–			

A total of 31 serum samples were collected from subjects (18 male and 13 female) with wounds but no signs of disease, heterotopic ossification negative (HO−). A total of 10 serum samples were collected from heterotopic ossification positive (HO+) subjects (9 male and 1 female). Serum samples were collected at time of surgery. The surgical procedure (or wound) differed between subjects, with the most common procedures being total hip arthroplasty (THA), open reduction and internal fixation, hip revision, and HO excision. Subjects ranged in age from 22 to 83 years old

**Fig. 2:**
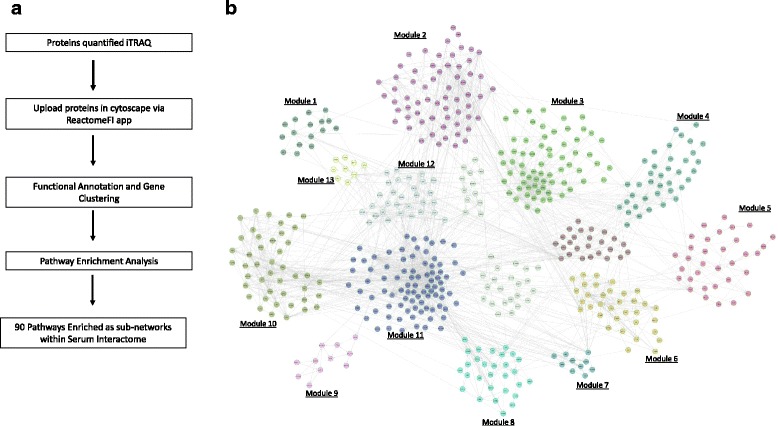
ReactomeFI analysis of iTRAQ data from serum samples. **a** Workflow pathway enrichment analysis iTRAQ data. All proteins quantified in the iTRAQ experiment, regardless of the disease state, were uploaded in cytoscape via ReactomeFI application. Genes were clustered based on pathway and sub-network annotations. A pathway enrichment analysis indicated that 90 pathways were enriched within the proteomic space of serum samples. **b** Reactome analysis serum samples. Proteins were clustered using ReactomeFI, and 13 major modules were identified. A pathway enrichment analysis was completed for each module using an FDR >0.01

**Fig. 3:**
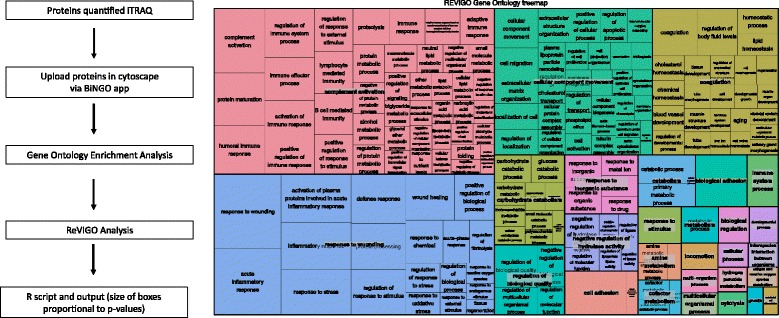
Gene ontology enrichment analysis iTRAQ data. All proteins quantified in the iTRAQ experiment regardless of the disease state were uploaded into cytoscape via BiNGO. BiNGO results were analyzed via REVIGO (reduce and visualize gene ontology)

The interactome (Fig. 2) and subsequent pathway enrichment analysis (Table 2) indicated extracellular matrix organization, ECM-receptor interaction, response to elevated platelet cytosolic Ca^+2^, and complement and coagulation cascades were enriched. The gene ontology biological process enrichment analysis included response to wounding, acute inflammatory response, and activation of plasma proteins involved in acute inflammatory response as top enriched biological processes (Fig. 3; and Additional file 2).

**Table 2:** Pathway enrichment summary iTRAQ data

Biological pathway	Pathway database	Ratio	Pathway proteins	Dataset proteins	*p* value	FDR
Extracellular matrix organization	R	0.0243	248	64	1.11E−16	1.30E−14
ECM-receptor interaction	K	0.0085	87	31	1.11E−16	1.30E−14
Response to elevated platelet cytosolic Ca^2+^	R	0.0081	83	45	1.11E−16	1.30E−14
Complement and coagulation cascades	K	0.0068	69	46	1.11E−16	1.30E−14
Beta1 integrin cell surface interactions	N	0.0065	66	27	1.11E−16	1.30E−14
Formation of fibrin clot (clotting cascade)	R	0.0038	39	24	1.11E−16	1.30E−14
Focal adhesion	K	0.0203	207	37	8.46E−12	8.46E−10
Staphylococcus aureus infection	K	0.0054	55	18	2.67E−10	2.35E−08
Beta3 integrin cell surface interactions	N	0.0042	43	16	4.32E−10	3.37E−08
L1CAM interactions	R	0.0077	79	20	2.15E−09	1.38E−07

All proteins quantified in the iTRAQ experiment, regardless of the disease state, were uploaded in cytoscape via ReactomeFI application. A pathway enrichment analysis was executed for the entire reactome

*R* Reactome, *K* KEGG, *N* NCI PID

In total, 10 candidate proteins were selected for SRM-MS from the iTRAQ studies based on relative fold changes in iTRAQ ratios and the characteristics of the proteotypic peptides for each protein (Table 3). Eight proteins, osteomodulin (OMD), collagen alpha-2(l) chain precursor (COL1A2), collagen alpha-1(V) chain isoform 2 preprotein (COL5A1), alkaline phosphatase (ALPL), phosphatidate phosphatase LPIN2 (LPIN2), RRP12-like protein (RRP12), TRAF3-interacting protein 1 (TRAF3), and protein phosphatase 1J (PPM1J), were selected from the serum iTRAQ results and subsequent bioinformatics analysis. Two additional proteins, bone sialoprotein 2 precursor (IBSP) and osteocalcin preprotein (BGLAP), were selected from an iTRAQ survey of tissue samples from HO-positive and HO-negative samples from the same study subjects because the ratio reported from the tissue data suggested a significant increase of this protein in the disease state compared to non-disease (manuscript in preparation).

**Table 3:** iTRAQ proteomics directed SRM candidate selection

Gene	GI accession number	Protein	Gene ontology: molecular function	Gene ontology: cellular component	Peptide ID	Peptide sequence
IBSP	167466187	Bone sialoprotein 2 precursor [*Homo sapiens*]		Extracellular matrix [GO:0031012]; extracellular region [GO:0005576]; extracellular space [GO:0005615]; membrane [GO:0016020]; membrane-bounded vesicle [GO:0031988]	SRM-1 SRM-2 SRM-3	HAYFYPHLK IEDSEENGVFK AYEDEYSYFK
BGLAP	40316933	Osteocalcin preproprotein [*Homo sapiens*]	Calcium ion binding [GO:0005509]; hydroxyapatite binding [GO:0046848]; structural constituent of bone [GO:0008147]; structural molecule activity [GO:0005198]	Cytoplasm [GO:0005737]; dendrite [GO:0030425]; endoplasmic reticulum lumen [GO:0005788]; extracellular space [GO:0005615]; Golgi lumen [GO:0005796]; membrane-bounded vesicle [GO:0031988]; perikaryon [GO:0043204]; rough endoplasmic reticulum [GO:0005791]	SRM-4 SRM-5 SRM-6	YLYQWLGAPVPYPDPLEPR GAAFVSK QEGSEVVK
OMD	4826876	Osteomodulin precursor [*Homo sapiens*]		Extracellular exosome [GO:0070062]; extracellular region [GO:0005576]; extracellular space [GO:0005615]; Golgi lumen [GO:0005796]; lysosomal lumen [GO:0043202]; proteinaceous extracellular matrix [GO:0005578]	SRM-7 SRM-8 SRM-9	IDYGVFAK LLLGYNEISK NLEHLYLQNNEIEK
COL1A2	48762934	Collagen alpha-2(I) chain precursor [*Homo sapiens*]	Extracellular matrix structural constituent [GO:0005201]; heparin binding [GO:0008201]; integrin binding [GO:0005178]; metal ion binding [GO:0046872]; platelet-derived growth factor binding [GO:0048407]; proteoglycan binding [GO:0043394]	Basement membrane [GO:0005604]; collagen type V trimer [GO:0005588]; endoplasmic reticulum lumen [GO:0005788]; extracellular exosome [GO:0070062]; extracellular matrix [GO:0031012]; extracellular region [GO:0005576]	SRM-10 SRM-11 SRM-12	GPAGPSGPAGK GEIGNPGR AQPENIPAK
COL5A1	495528154	Collagen alpha-1(V) chain isoform 2 preproprotein [*Homo sapiens*]	Extracellular matrix structural constituent [GO:0005201]; heparin binding [GO:0008201]; integrin binding [GO:0005178]; metal ion binding [GO:0046872]; platelet-derived growth factor binding [GO:0048407]; proteoglycan binding [GO:0043394]	Basement membrane [GO:0005604]; collagen type V trimer [GO:0005588]; endoplasmic reticulum lumen [GO:0005788]; extracellular exosome [GO:0070062]; extracellular matrix [GO:0031012]; extracellular region [GO:0005576]	SRM-13 SRM-14 SRM-15	VLDFHNLPDGITK DAQLSAPTK QLYPASAFPEDFSILTTVK
ALPL	530360994	Predicted: alkaline phosphatase, tissue-nonspecific isozyme isoform X3 [*Homo sapiens*]	Alkaline phosphatase activity [GO:0004035]; metal ion binding [GO:0046872]; pyrophosphatase activity [GO:0016462]	Anchored component of membrane [GO:0031225]; extracellular exosome [GO:0070062]; extracellular membrane-bounded organelle [GO:0065010]; extracellular space [GO:0005615]; integral component of membrane [GO:0016021]; membrane [GO:0016020]; plasma membrane [GO:0005886]; proteinaceous extracellular matrix [GO:0005578]	SRM-16 SRM-17 SRM-18	GFFLLVEGGR ANEGTVGVSAATER LDGLDLVDTWK
LPIN2	530425009	Predicted: phosphatidate phosphatase LPIN2 isoform X2 [*Homo sapiens*]	Phosphatidate phosphatase activity [GO:0008195]; transcription coactivator activity [GO:0003713]	Cytosol [GO:0005829]; endoplasmic reticulum membrane [GO:0005789]; nucleus [GO:0005634]	SRM-19 SRM-20 SRM-21	GLEPEVAALYFPK VDSPSK VIPSEDNLISEVEK
RRP12	547234776	RRP12-like protein isoform 3 [*Homo sapiens*]	Poly(A) RNA binding [GO:0044822]	integral component of membrane [GO:0016021]; nuclear membrane [GO:0031965]; nucleolus [GO:0005730]	SRM-22	FGFELVK
					SRM-23	AAQHGVCSVLK
					SRM-24	AVEEGLTYK
TRAF3	578804053	Predicted: TRAF3-interacting protein 1 isoform *X*2 [Homo sapiens]			SRM-25 SRM-26 SRM-27	YLHDIITEVIR ISFLQK ITDCAVEPLK
PPM1J	65506328	Protein phosphatase 1J [*Homo sapiens*]	Protein serine/threonine phosphatase activity [GO:0004722]		SRM-28 SRM-29 SRM-30	AIIVR ASFSRPTFLQLSPGGLR SPDLPNAASAPPAAAPEAPR

Ten proteins were chosen for selected monitoring reaction (SRM) MS analysis based on iTRAQ data. Three peptides per protein were synthesized for assay development, and the molecular function and cellular component gene ontologies for each SRM candidate were annotated

### SRM analysis

SRM transition parameters of all 30 peptides targeting the top overexpressed proteins quantified by iTRAQ were incorporated into a SRM-MS assay method. Each protein was independently quantified by 3 peptides in 30 individual patient serum samples. Seventeen peptides representing 9 of the 10 proteins from the initial experimental design, alkaline phosphatase, osteocalcin, alpha-2 type I collagen, collagen alpha-1(V) chain isoform 2 preprotein, bone sialoprotein 2, osteomodulin, protein phosphatase IJ, and RRp12-like protein, were validated as SRM targets after successfully being detected and quantified in serum (Additional file 3). Phosphatidate phosphatase LPIN2 isoform X2 was detectable only by one peptide in five HO− and five HO+ samples and was dropped from the panel.

**Fig. 4:**
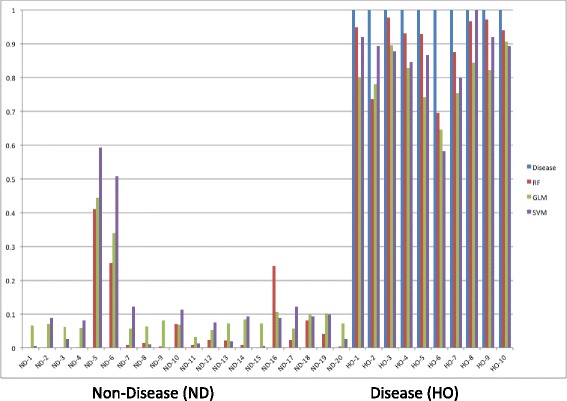
Summary of model comparison SRM assay. Samples were annotated as either disease state (heterotopic ossification positive—HO+) or non-disease (ND) state (heterotopic ossification negative—HO−). Three different statistical models were utilized to analyze the SRM-MS data: random forest (RF; *red line*), generalized linear model (GLM; *green line*), and support vector machine learning (SVM; *purple line*). A non-disease state for HO− prediction was 0 (*blue line left panel*) and a disease state for HO+ prediction was 1 (*blue line right panel*). All three statistical models performed similarly

**Fig. 5:**
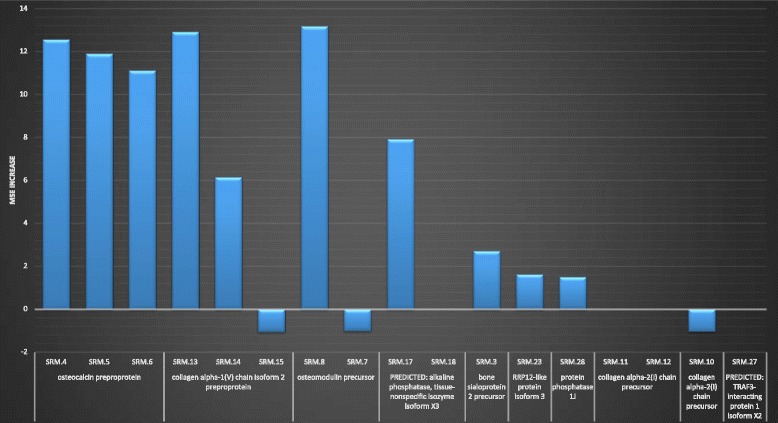
Mean square error analysis for random forest model. Using the random forest (RF) model, peptides with a mean square error (MSE) increase >8 were considered important variables because random permutation of these variables had a significant impact on the model prediction of disease state versus non-disease state

The RF, GLM, and SVM algorithms produced predictive models that were comparable in performance (Fig. 4), but RF predictions were closest to the true disease state for 9 of the 10 HO+ subjects (binary value where *0* indicates HO negative or non-disease (ND) and *1* indicates HO positive or disease (D)) (Fig. 4). Using the RF generated model, the SRM peptides were ranked by the mean square error (MSE) increase if the peptide is randomly permuted. If a peptide is an important predictor, then the model fit decreases when it is randomly permuted and the overall MSE increases. Peptides SRM-8, SRM-13, SRM-4, SRM-5, and SRM-6 representing proteins osteocalcin preprotein, osteomodulin precursor, and collagen alpha-1(v) chain isoform 2 preprotein were identified as potential biomarkers for HO (Fig. 5).

**Fig. 6:**
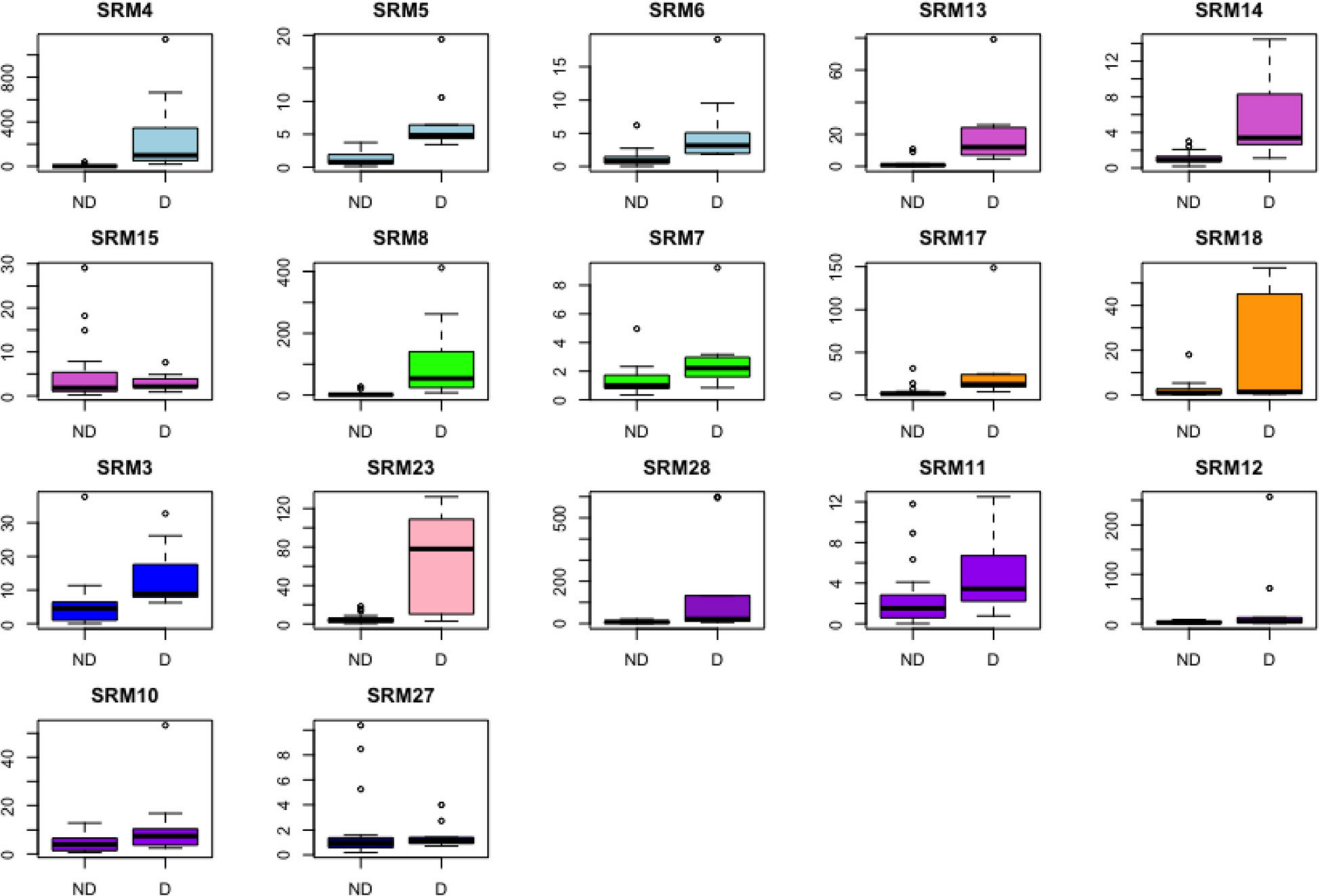
Box-whisker plots for selection reaction monitoring peptide candidates for heterotopic ossification (HO+/D) and non-disease (HO−/ND) serum samples. Distribution of selection reaction monitoring (SRM) normalized abundance ratios for each peptide for heterotopic ossification negative samples (non-disease; ND) and heterotopic ossification positive samples (disease; D)

Relative expression levels of the peptide biomarkers were significantly different for SRM peptides, SRM-4, SRM-5, SRM-6, SRM-13, SRM-14, SRM-8, SRM-3, SRM-23, derived from osteocalcin, collagen alpha-1(V) chain, osteomodulin, bone sialoprotein 2, and RRP12-like protein (Fig. 6). Three osteocalcin peptides (SRM4, 5, and 6) had similar patterns between the disease and non-disease state, supporting a higher abundance in HO+ compared to HO−. Collagen alpha 1 (SRM13, 14, 15) had two peptides (SRM13 and 14) that support this protein in higher abundance in HO, whereas SRM15 showed no difference between HO− and HO+. Two peptides were measured for osteomodulin (SRM7 and 8), and both peptides were elevated in HO+ compared to HO−. Two peptides were measured for alkaline phosphatase (SRM17 and 18), and SRM18 had greater dispersion in the upper quartile in HO+ compared to HO−. A single peptide was measured for bone sialoprotein (SRM3), and the data support higher peptide abundance in HO+ compared to HO−. A single peptide was measured for RRP12 (SRM 23) and demonstrated greater dispersion in expression in HO+ versus HO− samples. One peptide was quantified for TRAF3 (SRM27), which showed no difference between HO+ and HO−.

**Fig. 7:**
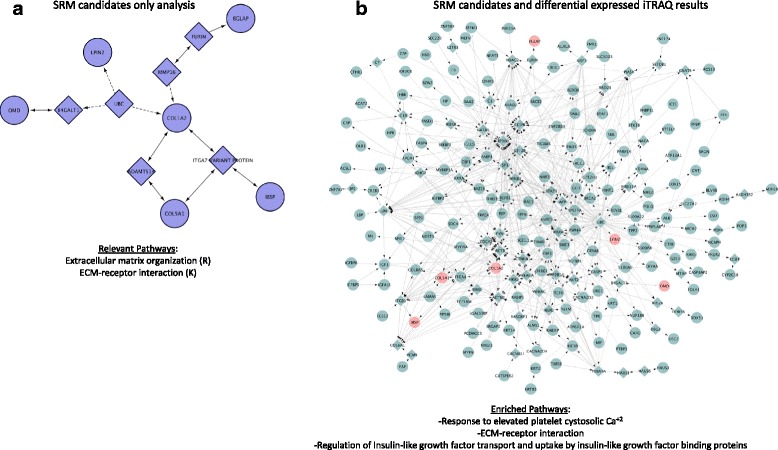
Summary target candidates for selection reaction monitoring assay. **a** The 10 proteins used in the selection reaction monitoring (SRM) assay were analyzed via ReactomeFI in cytoscape. Six of the candidates (*circles*) were clustered with six linker genes (*diamonds*). Relevant pathways for wound healing and ossification within this small interactome were extracellular matrix organization and extracellular matrix-receptor interaction. **b** The 10 proteins (*red nodes*) used in the selection reaction monitoring (SRM) assay were analyzed via ReactomeFI in cytoscape with all proteins that were differentially regulated (*green nodes*) in the iTRAQ experiment

Protein-protein interactions between SRM candidates were identified by ReactomeFI (Fig. 7) such that their connections with each other (panel a) and within the context of differentially regulated proteins in iTRAQ experiment (panel b) could be analyzed. Of interest, six of the SRM candidates, BGLAP, COL1A2, COL5A1, IBSP, LPIN2, and OMD, were clustered together using linker regions and are involved in extracellular matrix organization and ECM-receptor interaction (panel a). When these candidates were examined in conjunction with other differentially regulated proteins, response to elevated platelet cystosolic Ca^+2^ sub-network was enriched (panel b). Collectively, these data suggest that there are several sub-networks, which are highly connected through protein-protein interactions that contain proteins that are differentially expressed in HO.

## Discussion

The presence of HO is determined using radiographs, which limits the ability to predict patients that are susceptible to HO and complicates early diagnosis of the disease as aberrant bone formation must first be detectable. Furthermore, using tissue samples to identify early signs of HO can be difficult because the proteome of tissue within a wound bed can vary widely depending on location within the wound bed [21]. Using tissue for disease identification requires invasive sampling and like all biopsy results, is dependent on the location of the sample in relation to the suspected disease foci.

A biomolecular screening tool using serum from wounded patients could allow for earlier diagnosis, intervention, and the potential development of novel therapeutics, to prevent development of HO [2, 9]. A major challenge with identifying systemic markers is the need for data-driven approaches. Applying a priori knowledge limits the advancement of screening assays because the majority of protein candidates, for example, MMPs are involved in normal healing and disease processes [22]. Effective diagnostic panels require multiple biomarkers across different gene families because the disease state is more often a consequence of misregulation of protein expression rather than a single mutation of a critical protein.

Another challenge for devising a pharmaceutical treatment for HO is the lack of knowledge regarding metabolic processes and misregulated cellular signaling events underlying the disease. Since HO has similar characteristics as seen in the normal physiology of fracture healing, treatment options for HO need to be very specific to avoid impairment of normal bone healing [22]. Identifying biomarkers that allow for early identification of HO is confounded by the active and ongoing inflammatory response present due to injury. During the inflammatory phase of the wound healing process, without the formation of heterotopic ossification, there will be a strong signal in the biological space of proteins related to wound healing.

Utilizing a shotgun proteomics assay, iTRAQ, qualitative expression levels were determined for all detectable proteins (1220) from serum samples collected from HO+ and HO− subjects and used to identify proteins that are differentially regulated between the disease and non-disease state. This global proteomics approach enabled a data-driven methodology. The bioinformatics analyses built networks of functionally related proteins capable of identifying crosstalk through protein-protein interactions between sub-networks. The goal of this approach was to identify biomarkers, proteins linked to misregulated pathways and that are differentially expressed in the disease state compared to non-disease state. The current research found that serum from both healthy and disease patients is enriched for proteins involved in the response to elevated platelet Ca^+2^, wound healing, and extracellular matrix organization, and that these pathways include proteins that are differentially regulated in the disease state (Table 2).

Shotgun proteomic techniques including iTRAQ provide a knowledgebase for identifying potential clinical biomarkers without the need for a priori knowledge, but results are semi-quantitative and require follow-up validation using a quantitative assay. To transition the semi-quantitative iTRAQ results into a clinical diagnostic system, we developed and utilized SRM-MS assays to precisely and robustly quantify 10 proteins chosen based on expression ratios from the iTRAQ experiment combined with functional annotations, including gene ontology and pathway information. Using a random forest model and the SRM-MS data, osteocalcin preproprotein, osteomodulin precursor, and collagen alpha-1(V) chain isoform 2 preprotein were determined to be the best candidates for predicting the disease state (HO+).

The model predictions of these targets as diagnostic markers are supported by a study of osteoclast and osteoblast activity after total hip arthroplasty, which found that osteocalcin increased in individuals who developed HO [23]. Osteocalcin (gene BGLAP; P02818) is secreted by bone-forming osteoblasts [24], and a strong overexpression of osteocalcin mRNA in HO isolated cells has been observed [25]. The wound fluid from blast-injured patients has osteoinductive signaling properties [5]. Serum from patients with TBI induced an increase in skeletal muscle cells, and the high levels of alkaline phosphatases suggested an increased osteogenic capability [10].

Bone formation and remodeling require a balance between osteoclast and osteoblast activity [26]. Osteomodulin (OMD), or osteoadherin, is part of the leucine-rich repeat proteins (SLRPs) located in the extracellular matrix. OMD is expressed by osteoblasts and is involved in the regulation of bone formation [27]. OMD has also been shown to regulate the diameter and shape of collagen fibrils [28]. The SRM findings presented here for both osteocalcin and osteomodulin in HO are consistent with the cell data from resected human HO bone that expressed the osteoblast phenotype (type I collagen) [29]. Other investigators have reported that collagen expression was increased in tissue from wounds with HO for COL10A1, COL4A3, and COL11A1 [4, 30].

## Conclusions

This study is the first reported SRM-MS analysis of serum from individuals with and without heterotopic ossification. Differences in the serum proteomic profile between healthy and diseased subjects were identified. Furthermore, our results indicate that normal wound healing signals can impact the ability to identify biomarkers, and a multi-protein panel assay, including osteocalcin preproprotein, osteomodulin precursor, and collagen alpha-1(v) chain isoform 2 preprotein, may provide a solution for HO detection and monitoring. The proteomic analysis within this report focuses on protein abundance, ignoring protein post-translational modifications (PTM). Of interest, osteocalcin has several amino acid residues that are susceptible to PTM that influence the function of this protein [31]. Future studies are planned to identify the presence and potential of differentially regulated PTMs in HO.

## Additional files

Additional file 1: Selection reaction monitoring (SRM) peptide transition parameters for protein candidates. Transition parameters and retention times of the 30 peptides were confirmed individually using an Agilent 6495 Triple Quadrapole Mass Spectrometer for both doubly and triply charged precursor ions. Five or 6 transitions per peptide precursor were selected for SRM analysis. In total, 350 transitions were optimized to identify and quantify 30 peptides. SRM target protein names, representative proteotypic peptide sequences, and SRM transition parameters are provided. (XLSX 68 kb)

Additional file 2: iTRAQ serum gene ontology enrichment analysis. All proteins quantified in the serum via iTRAQ were analyzed using BiNGO and cytoscape on June 23, 2016. An over-representation analysis, hypergeometric test with a Benjamini & Hochberg False Discovery Rate (FDR) correction, was executed with a significance level of <0.05. (XLSX 130 kb)

Additional file 3: Scatterplots selection reaction monitoring assay. Plot matrix of SRM peptide abundance in blood serum from heterotopic positive (blue) and negative (gold) subjects. (PNG 3461 kb)

## Abbreviations

ALPL: Alkaline phosphatase
AUC: Area under the curve
BAMC: Brooke Army Medical Center
BGLAP: Osteocalcin preprotein
COL1A2: Collagen alpha-2(l) chain precursor protein
COL5A1: Collagen alpha-1(V) chain
FDA: Food and Drug Administration
GLM: Generalized linear model
HO: Heterotopic ossification
HO−: Heterotopic ossification, disease negative
HO+: Heterotopic ossification, disease positive
HPLC: High-performance liquid chromatography
IBSP: Bone sialoprotein 2 precursor
IRB: Institutional Review Board
iTRAQ: Isobaric tags for relative and absolute quantitation
KEGG: Kyoto Encyclopedia of Genes and Genomes
LPIN2: Phosphatidate phosphatase
MMP: Matrix metalloproteinase
MS: Mass spectrometry
MSE: Mean square error
ND: Non-disease
NSAID: Nonsteroidal anti-inflammatory drug
OEF: Operation Enduring Freedom
OIF: Operation Iraqi Freedom
OMD: Osteomodulin
PPM1J: Protein phosphatase 1J
PTM: Post- translational modifications
RF: Random forest
ROC: Receiver operating characteristic
RRP12: RRP12-like protein
SCI: Spinal cord injury
SRM-MS: Selection reaction monitoring mass spectrometry
SVM: Support vector machine learning
TBI: Traumatic brain injury
TCEP: Tris-(2-carboxyethyl) phosphine
TFA: Trifluoroacetic acid
TRAF3: TRAF3-interacting protein 1
USAMRMC: US Army Medical Research and Materiel Command
