# Supplementary figures and images for: A survey of proteomic biomarkers for heterotopic ossification in blood serum
Source: J Orthop Surg Res. 2017 May 4;12:69. doi: 10.1186/s13018-017-0567-2 (PMC5418723; doi:10.1186/s13018-017-0567-2)

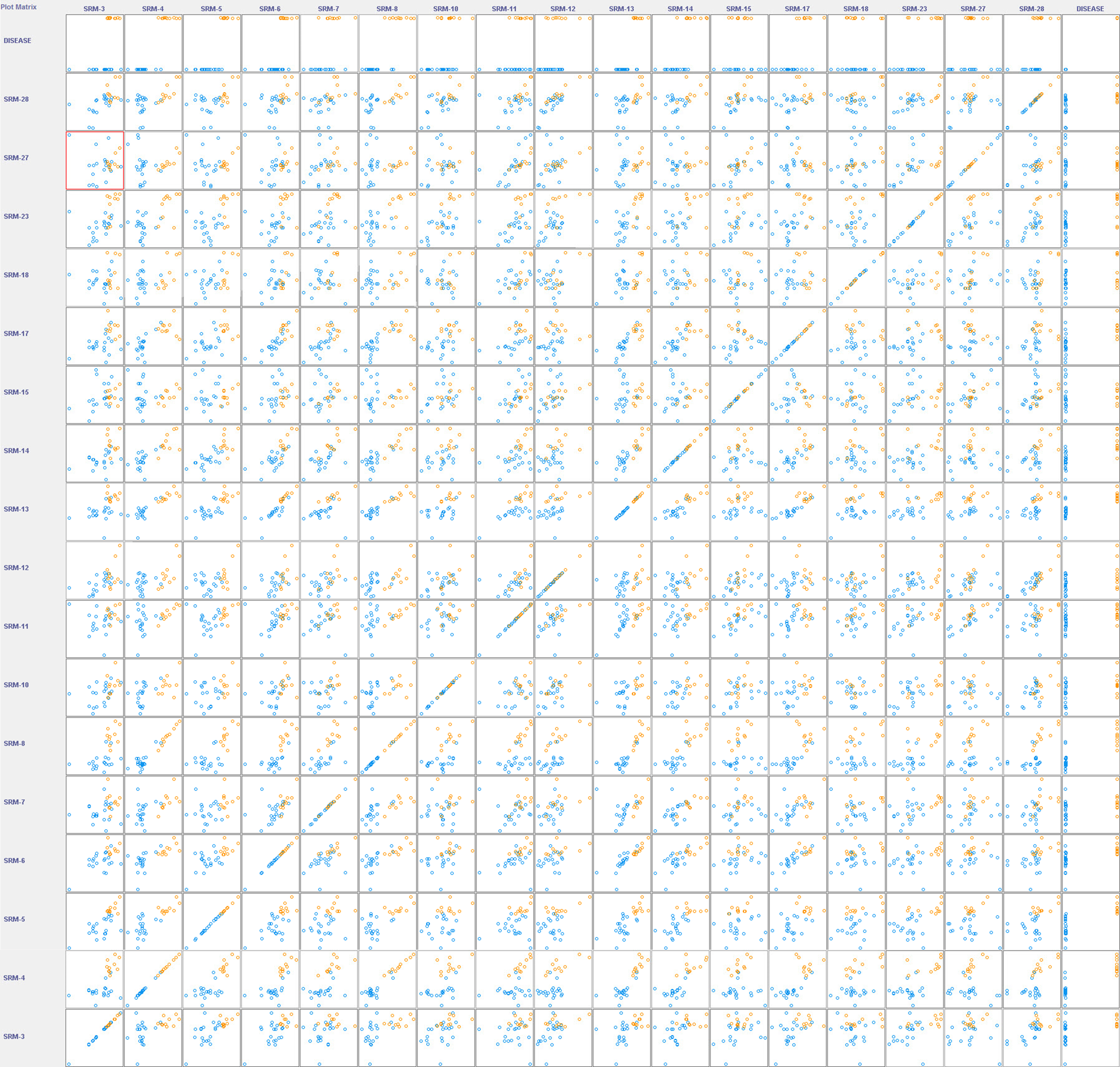

Supplement: Supplementary file 3 — Scatterplots selection reaction monitoring assay. Plot matrix of SRM peptide abundance in blood serum from heterotopic positive (blue) and negative (gold) subjects. (PNG 3461 kb) [file 13018_2017_567_MOESM3_ESM.png]
